# Process modeling of municipal solid waste compost ash for reactive red 198 dye adsorption from wastewater using data driven approaches

**DOI:** 10.1038/s41598-021-90914-z

**Published:** 2021-06-02

**Authors:** Mohammad Hadi Dehghani, Mehdi Salari, Rama Rao Karri, Farshad Hamidi, Roghayeh Bahadori

**Affiliations:** 1 Department of Environmental Health Engineering, School of Public Health, Tehran University of Medical Sciences, Tehran, Iran; 2 Institute for Environmental Research, Center for Solid Waste Research, Tehran University of Medical Sciences, Tehran, Iran; 3 Student Research Committee, Department of Environmental Health Engineering, School of Public Health, Hamadan University of Medical Health, Hamadan, Iran; 4 Petroleum and Chemical Engineering, Faculty of Engineering, Universiti Teknologi Brunei, Gadong, Brunei Darussalam

**Keywords:** Environmental sciences, Chemistry

## Abstract

In the present study, reactive red 198 (RR198) dye removal from aqueous solutions by adsorption using municipal solid waste (MSW) compost ash was investigated in batch mode. SEM, XRF, XRD, and BET/BJH analyses were used to characterize MSW compost ash. CNHS and organic matter content analyses showed a low percentage of carbon and organic matter to be incorporated in MSW compost ash. The design of adsorption experiments was performed by Box–Behnken design (BBD), and process variables were modeled and optimized using Box–Behnken design-response surface methodology (BBD-RSM) and genetic algorithm-artificial neural network (GA-ANN). BBD-RSM approach disclosed that a quadratic polynomial model fitted well to the experimental data (F-value = 94.596 and R^2^ = 0.9436), and ANN suggested a three-layer model with test-R^2^ = 0.9832, the structure of 4-8-1, and learning algorithm type of Levenberg–Marquardt backpropagation. The same optimization results were suggested by BBD-RSM and GA-ANN approaches so that the optimum conditions for RR198 absorption was observed at pH = 3, operating time = 80 min, RR198 = 20 mg L^−1^ and MSW compost ash dosage = 2 g L^−1^. The adsorption behavior was appropriately described by Freundlich isotherm, pseudo-second-order kinetic model. Further, the data were found to be better described with the nonlinear when compared to the linear form of these equations. Also, the thermodynamic study revealed the spontaneous and exothermic nature of the adsorption process. In relation to the reuse, a 12.1% reduction in the adsorption efficiency was seen after five successive cycles. The present study showed that MSW compost ash as an economical, reusable, and efficient adsorbent would be desirable for application in the adsorption process to dye wastewater treatment, and both BBD-RSM and GA-ANN approaches are highly potential methods in adsorption modeling and optimization study of the adsorption process. The present work also provides preliminary information, which is helpful for developing the adsorption process on an industrial scale.

## Introduction

In parallel with rapid population increase and high urbanization and industrialization rate, the concerns related to the release of various pollutants to many groundwater resources have grown dramatically around the world in the last decades^1^. Many industries like paper printing, textile dyeing, and other sectors such as photography, pharmaceuticals, food, and cosmetic industries annually generate wastewaters containing a wide variety of synthetic aromatic dyes in large quantity^2^. Among different types of water pollutants, synthetic dyes are regarded as an important toxic group for humans, the fauna and flora, even at a low level of exposure^3^. Therefore, human health and environmental problems make the efficient treatment of these types of wastewaters so necessary. So far, different processes have been investigated and developed for industrial wastewater treatment. Although biological processes have attracted significant interest as an economical and efficient treatment method, those are faced with several problems of the grown death rate of bacteria population exposed to the high toxicity wastewaters and hard biodegradability of the dye wastewaters^4^. Recently, advanced oxidation processes have received great attention as alternative treatments for recalcitrant wastewaters. However, the production of toxic by-products remains a major disadvantage of these methods^5^. The adsorption process has received wide attention for efficient treatment of different water matrices due to its simplicity, economic feasibility, regulatory compliance, public acceptance, and environmentally friendly procedure^6^. Availability, cheapness, high efficiency, and environmentally friendly user, easy to use, easy operation and non-susceptibility of an adsorbent to pollutants can potentially encourage authorities to consider the adsorption process as one of the most deserved treatment technique^7^. In spite of the abundant application of activated carbon as an efficient adsorbent due to its virtues such as superior adsorption capacity, large specific surface area, high production cost and reuse-related drawback causes that the environmental researchers make more effort to find new efficient adsorbent without shortcomings coming from activated carbon^8^. Composting is an efficient, sustainable, and cheap solid waste management approach is used for the biological degradation of biodegradable organic wastes in aerobic conditions^9^. In some study, fly ash derived from various sources has attracted significant interest as novel adsorbents for the uptake of various pollutants from aquatic environments, including date seed ash^10^, activated fly ash^11^, coal fly ash^12,13^, fly ash zeolite^14^, and municipal solid waste incineration fly ash^15^. No study was reported in the literature to investigate the adsorptive ability of ash derived from the combustion of municipal solid waste (MSW) compost. Therefore, the present study aimed to investigate the adsorptive capability of MSW compost ash for RR198 dye in synthetic wastewater. Under the treatment process, the process parameters, namely initial pH of the solution, contact time, adsorbent dosage, and initial dye concentration, were modeled and optimized by Box–Behnken design-response surface methodology (BBD-RSM) and artificial neural network (GA-ANN). RSM technique estimates the possible correlation between the input parameters and dependent parameter, revealing quadratic and interactive effects of independent variables on a response, in parallel to the linear effect estimation, and reduces the replicate of experiments, and subsequently material cost and consuming time^16^. ANN approach is a robust mathematical tool for modelling different behaviors, even the complex non-linear relationship. Unlike RSM, ANN does not require necessarily any experimental design, so that this approach can be successful to model any informal set of the experimental data^17,18^. In the following, isotherm, kinetic, and thermodynamic characteristics of the RR198 dye absorption onto MSW compost ash, and reusability test of MSW compost ash were performed at the optimal condition of the process parameters. The other objectives were to characterize MSW compost ash by some analyses such as SEM, XRD, XRF, BET-BJH. Additionally, carbon content and CHNS analyses were done for MSW compost ash.

## Materials and method

### Materials

MSW compost was supplied by the waste management organization of Tehran municipality, Iran. RR198 was purchased from Alvan Sabet Company (Hamedan, Iran). The properties of the compost supplied were tabulated in Table 1.

**Table 1:** Properties of the MSW compost.

Sample	MSW compost
Organic matter content (%)	68.8 ± 0.4
Dry matter content (%)	77.1 ± 0.7
pH	8.14 ± 0.17
Electrical conductivity (mS cm^−1^)	2.57 ± 0.4
Respiration index (mg O_2_ g^−1^ OM h^−1^)	1.67
Bulk density (kg L^−1^)	0.42
Air-filled porosity (%)	59
Nitrogen Kjeldahl (%, dry basis)	1.98 ± 0.13
C/N ratio	16
*E. coli* (CFU g^−1^)	< 10

All the other chemicals and solvents used in this study were of analytical reagent grade and obtained from Merck Company. MSW compost ash was produced by keeping MSW compost under 550 °C temperature for 4 h in a furnace. Thereafter, compost ash was sieved below 20 mesh and stored in a bottle for further application^6^.

### Experimental procedure

All the adsorption experiments were conducted in batch mode in a 500 mL sample volume. The effect of independent variables, namely initial pH of the solution, reaction time, MSW compost ash loading and initial RR198 concentration at the range and levels presented in Table 1, was investigated on the efficiency of RR198 uptake by MSW compost ash. Further, RR198 adsorption isotherm, kinetic and thermodynamic studies were performed based on experimental data at optimal condition. The initial pH of the medium was regulated by 0.1 N NaOH and 0.1 N H_2_SO_4_ solution, and the solution temperature was set by incubator shaker for thermodynamic studies. After each experiment, the MSW compost ash was isolated from the sample by centrifuging at 10,000 rpm for 15 min. DR 5000 spectrophotometer was employed to determine the remaining concentration of RR198 at a maximum absorptive wavelength of 518 nm^19^. All the adsorption experiments were conducted triplicate, and the results were reported as average values.

### Characterization techniques for MSW compost ash

In order to evaluate surface characteristics of MSW compost ash, SEM analysis (SEM, HITACHI S-4160, Japan) was used, and XRD analysis was used in order to assess crystalline properties of MSW compost ash using an X'Pert Pro diffractometer (Rigaku RINT2200, Japan) at scanning range from 10° to 80° at 40 kV with the electron probe current of 30 mA. The chemical composition of MSW compost ash was identified using the Philips XRF machine (Philips PW1480 model). Specific surface area and pore size distribution of MSW compost ash were analyzed by using BET/BJH analysis by Quanta chrome NOVA2000 automatic analyzer. The organic content of the sample was determined based on the ASTM D-2974 method^20^. CHNS-O Thermo Finnigan Elementary Analyzer Flash EA 1112 was employed to measure the total content of carbon, hydrogen, nitrogen, and sulphur via combustion of MSW compost ash in the presence of O_2_. The carbonate content of the sample was quantified by calcimetry in a Bernard calcimeter^21^.

### Experimental design, modeling, and optimization

In the present work, BBD experimental design approach was applied to provide data for process modeling in the range and level of the input parameters tabulated in Table 2. It was done in Design-Expert software.

**Table 2:** Experimental ranges and levels of the process parameters used in the Box–Behnken design.

Variable	Symbol	− 1	0	+ 1
pH	X_1_	3	7	11
Contact time(min)	X_2_	20	50	80
MSW compost ash dosage (g)	X_3_	0.5	1.25	2
RR198 concentration (mg L^−1^)	X_4_	20	60	100

The BBD-RSM approach was used to determine the relationships between the input variables and adsorption efficiency of RR198 by fitting the experimental data with a quadratic multivariate mathematical equation expressed as:

1 $$ {\text{Y}} = {\text{ a}}_{0} + \mathop \sum \limits_{{{\text{i}} = 1}}^{{\text{k}}} {\text{a}}_{{\text{i }}} {\text{x}}_{{\text{i}}} + { }\mathop \sum \limits_{{{\text{i}} = 1}}^{{\text{k}}} {\text{a}}_{{\text{ii }}} {\text{x}}_{{\text{i}}}^{2} { } + { }\mathop \sum \limits_{{{\text{i}} \ne 1}}^{{\text{n}}} {\text{a}}_{{{\text{ij}}}} {\text{x}}_{{\text{i}}} {\text{x}}_{{\text{j}}} + {\upvarepsilon } $$

Here, y is predicted value (%), a_0_ the constant-coefficient described as intercept, a_i_, a_ii_, a_ij_ stand for the linear, quadratic, and interaction regression coefficient, respectively, x_i_ and x_j_ correspond to the actual values of input variables, n and e are assigned to the input variable number and model error, respectively. Optimization of the input variables to maximize removal efficiency in the BBD-RSM technique was conducted based on a desirable function, in which the input factors were set in the fractional range, and the response was maximized^22–24^.

ANN approach was also used for modeling RR198 adsorption by MSW compost ash based on the same dataset. Multi-layered perception (MLP) is the most widely applied and researched neural network model, owing to its comparably simple algorithm and clear architecture^24–26^. In the present study, the MLP-ANN model constituted from Levenberg–Marquardt backpropagation training algorithm with three layers consisting of an input layer (four neurons), a hidden layer, and an output layer (one neuron), 1000 epochs, 1e−07 min-grad, and 1000 max-fail was used for process modeling. Tansig and purlin functions were used in the hidden layer and the last layer, respectively, as transfer functions. As predictive accuracy and performance of the ANN model are significantly affected by the number of hidden neurons, the best number of hidden neurons of the neural network was searched by means of empirical testing or trial and error. For this propose, the number of hidden neurons was varied from a minimum of 1 to a maximum of 20, and the optimal number was chosen based on the traditional measures of mean squared error (MSE) and determination coefficient (R^2^), which are determined as follows:

2 $$ {\text{MSE}} = \frac{1}{{\text{N}}}\mathop \sum \limits_{{{\text{i}} = 1}}^{{\text{N}}} \left( {\left| {{\text{z}}_{{{\text{p}},{\text{i}}}} - {\text{z}}_{{{\text{exp}},{\text{i}}}} } \right|} \right)^{2} . $$

3 $$ {\text{R}}^{2} = \frac{{\mathop \sum \nolimits_{{{\text{i}} = 1}}^{{\text{N}}} ({\text{z}}_{{{\text{p}},{\text{i}}}} - {\text{z}}_{{{\text{exp}},{\text{i}}}} ){ }}}{{\mathop \sum \nolimits_{{{\text{i}} = 1}}^{{\text{N}}} ({\text{z}}_{{{\text{p}},{\text{i}}}} - {\text{z}}_{{{\text{av}}}} )}}{ } $$

where z_p,i_ is the predicted value, z_exp,i_ and z_av_ show the experimental value and the mean of experimental values, and N is the number of experimental runs. The experimental design matrix used in the ANN model was the one used in the BBD-RSM model. The dataset was partitioned into three groups in a random manner, including training (75%), validation (15%), and test (15%) dataset. The dataset was normalized to 0.1–0.9 range by Eq. (4) to minimize the error. It causes that the training happens more efficiently^27^:

4 $$ {\text{y}}_{{\text{i}}} = 0.1 + 0.8{ } \times \frac{{{\text{x}}_{{\text{i}}} - {\text{ x}}_{{{\text{min}}}} }}{{{\text{x}}_{{{\text{max}}}} - {\text{ x}}_{{{\text{min}}}} }}{ } $$

Here, y_i_ stands for the normalized x_i_, and x_min_ the minimum value of x_i_, and x_max_ the maximum value of x_i_.

The prediction capability of the developed BBD-RSM and ANN models was finally evaluated by MSE and R^2^ statistics. GA approach was used to optimize the ANN model in MATLAB 2013 software. For this purpose, the developed model was written in a script file, and the fractional points were considered as the upper and lower levels of the independent factors. Finally, the optimization results of the process parameters based on BBD-RSM and GA-ANN approaches were compared to each other, and some control experiments were done to assess the accuracy of the results^7^.

## Results and discussion

### Characterization of MSW compost ash

**Figure 1:**
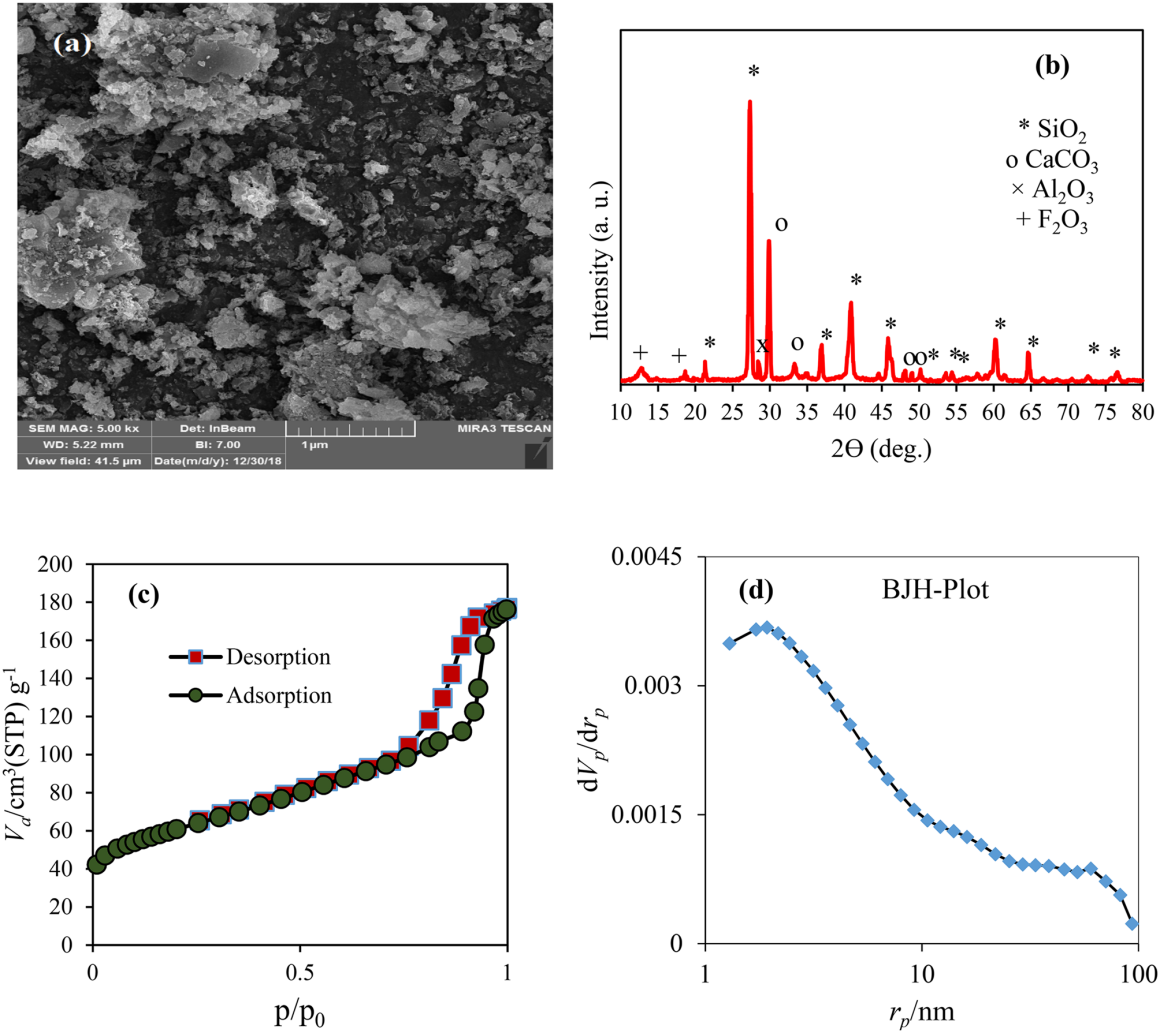
SEM (**a**), XRD (**b**), BET (**c**) and BJH analyses for CA.

Morphology, geometries, and structural properties of MSW compost ash samples were analyzed using SEM images with a magnification of ×50,000. Figure 1a shows the size distribution, surface morphology and particle shape of MSW compost ash. As can be seen, the synthesized sample is porous, coarse, angled and irregular in nature. Also, it is also found that the sample is fabricated from micro-nano multi-scale particles. The chemical composition of MSW compost ash was determined by the XRF technique. The corresponding result revealed that the most abundant oxide components in the sample structure are related to SiO_2_ (52.7), CaO (15.9%) and Al_2_O_3_ (7.9%). Also, a small portion of MSW compost ash is produced from Fe_2_O_3_, MgO, K_2_O, and TiO_2_. XRD patterns of MSW compost ash are given in Fig. 1b. As expected, the spectrum identified the major contribution of SiO_2_ (JCPDS Card ID 01-077-1066), CaCO_3_ (JCPDS Card ID 98-005-2151), and a minor contribution of crystalline phases of Al_2_O_3_ and Fe_2_O_3_ (JCPDS Card ID 98-001-6597) in the MSW compost ash sample, which is in a consistent with the findings observed in XRF analysis^21^. The result of the BET surface area of the MSW compost ash powder is displayed in Fig. 1c. The analysis showed a specific surface area of 50.14 m^2^/g and a total pore volume of 0.214 m^3^/g for the MSW compost ash sample. BJH test was also utilized to identify the pore size distribution of MSW compost ash. The related results, as displayed in Fig. 1d, illustrated that the pore size distribution of the material is mesoporous, where pore size mainly falls into a range from 2 to 50. The larger pore size can be assigned to intra-aggregate porosity, and the smaller pore size can be related to intra-particle porosity.

Table 3 shows the results of CNHS analysis for MSW compost ash. It was found that MSW compost ash contains carbon of 4.2%, hydrogen of 0.17%, nitrogen of 0.1%, and sulfur of 0.06%. The total organic matter content of MSW compost ash was determined to be about 6.48%, arising from incomplete combustion of the MSW compost. Also, the obtained carbonate content was measured to be about 5.61%.

**Table 3:** CNHS analysis and carbonate content of MSW compost ash.

Sample	Organic matter content (%)	Carbonate content (%)	%C	%H	%N	%S
CA	6.48 ± 0.14	5.61 ± 74	4.2 ± 0.01	0.17 ± 0.07	0.1 ± 0.009	0.06 ± 0.04

### Statistical analysis and model fitting

The constraints of independent variables, experimental removal efficiency along with the predicted removal efficiency of RR 198 by the BBD-RSM approach is presented in Table 4. Based on the statistical ANOVA analysis (see Table 5), suggested a reduced polynomial quadratic model. *P*-value > 0.05 was the criteria for the removal of some model terms from the developed model. The F-test and probability value statistics for the model were found to be 2543 and < 0001, respectively, representing that there exists only a 0.01% chance for F-value to be due to noise. It confirms the significance of the developed model. The higher values of R^2^ (close to 1) justify a strong correlation between the experimental and predicted values. The correlation coefficient, R^2^ was found to be 0.9755, indicating that only less than 3% of the total variation is not explained by the model. The results also show a reasonable agreement between Pred R^2^ (0.9436) and the Adj R^2^ (0.9652), which is quite satisfactory. It validates that the unnecessary variables are not included in the final model. The lack of fit value was calculated as 0.4961, which was most preferred. It is worthy to note that the value greater than 0.05 was not significant relative to the pure error^24^. The following mathematical equation expresses the developed model:

5 $$ \begin{aligned} {\text{Removal}}\,{\text{efficiency}} & = \, 74.0519 - 4.3443{\text{A}} + 0.2421{\text{B }} \\ & \quad + 9.1111{\text{C}} - 0.3244{\text{D}} - 0.0096{\text{AB }} \\ & \quad + 0.0075{\text{AD}} + 0.2162{\text{ A}}\widehat{{\text{A}}}^{2} + 0.0018{\text{B}}\widehat{{\text{A}}}^{2} \\ \end{aligned} $$

**Figure 2:**
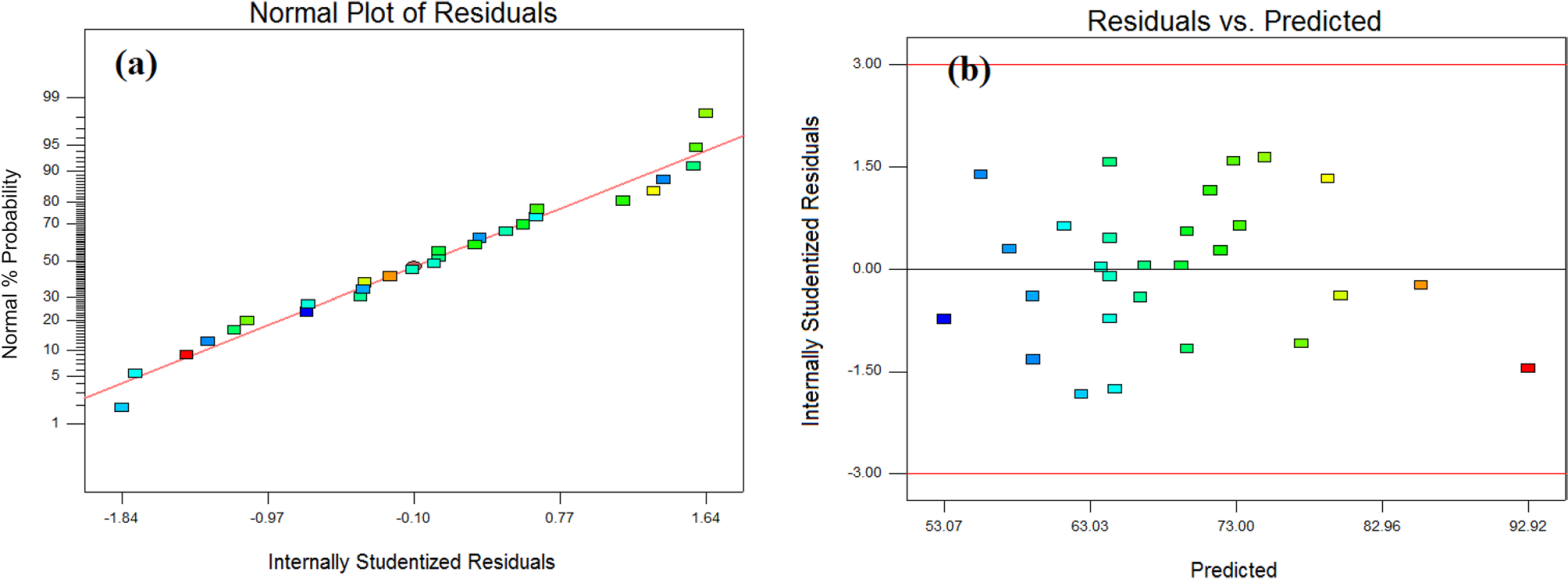
The normal plot of residual values (**a**), the diagnostic plot of residuals versus predicted values (**b**).

Diagnostic tools of normal plots were used to further verify the adequacy of the model. Figure 2a displays that the residual points are normally distributed around the straight line. Figure 2b shows a randomized distribution of residual points around the straight line, and the points do not follow a specific trend. To recognize the most effective parameters on the adsorption efficiency, the Pareto effect was calculated as follows^28^:

6 $$ {\text{P}}_{{\text{i}}} { }\left( {\text{\% }} \right) = \left[ {\frac{{({\text{b}}_{{\text{i}}}^{2} )}}{{\sum {\text{b}}_{{\text{i}}}^{2} }}} \right] \times 100 $$

**Figure 3:**
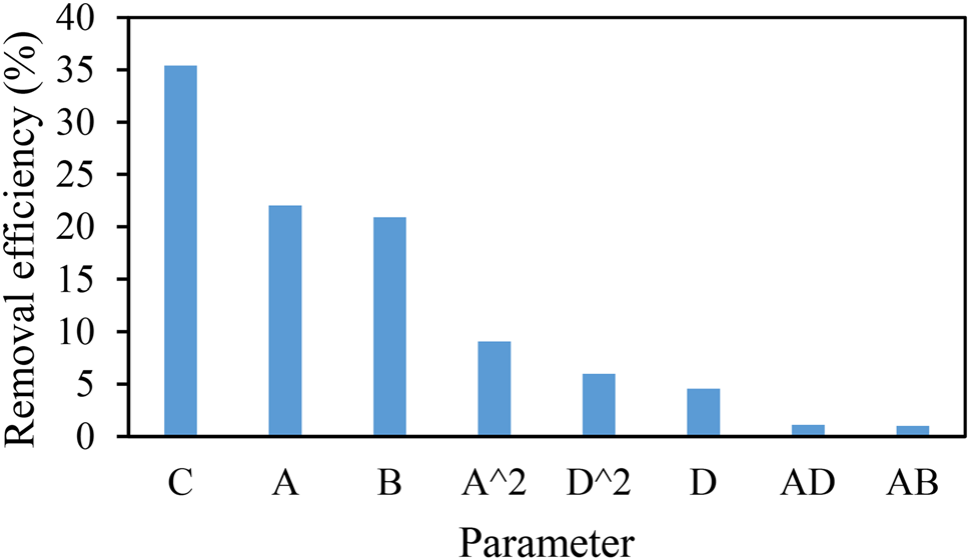
Pareto chart for adsorption of RR198 on MSE compost ash.

Here, Pi demonstrates the Pareto effect of each term included in the predicted model and b_i_ shows the regression coefficients from the regression equation in terms of coded values. Figure 3 indicates that the adsorbent dose, initial solution pH, and contact time present the highest impact on the adsorption efficiency, respectively.

**Table 4:** Box–Behnken design matrix and the experimental and response values of RR198 uptake by MSW compost ash.

pH	Time (min)	Adsorbent dose (mg L^−1^)	RR198 Con.	Experimental removal (%)	Predicted value (%) (BBD-RSM)	Predicted value (%) (GA-ANN)
3	20	0.5	20	65.9	66.45	65.87
11	20	2	100	66.8	66.74	64.92
7	50	1.25	20	70.4	69.64	69.54
3	80	0.5	20	81	79.25	80.96
7	80	1.25	60	67.8	69.63	67.72
7	50	2	60	73	71.21	72.87
11	80	0.5	20	63.8	63.77	64.25
7	50	0.5	60	58	57.54	58.30
3	20	2	100	74.9	72.82	75.04
11	80	0.5	100	62.1	61.27	60.49
7	50	1.25	100	62.3	64.74	61.90
11	20	0.5	20	57.4	55.57	57.38
7	50	1.25	60	65.1	64.38	64.66
11	20	2	20	69.3	69.24	69.30
3	80	2	20	91	92.92	90.62
11	20	0.5	100	52.1	53.07	55.75
11	80	2	20	76	77.44	74.55
3	20	0.5	100	57.4	59.15	57.48
3	50	1.25	60	74.1	73.23	75.71
11	50	1.25	60	59.9	62.45	59.50
3	20	2	20	79.6	80.12	79.58
11	80	2	100	77.1	74.94	70.76
3	80	0.5	100	72.3	71.95	72.20
7	20	1.25	60	58.5	59.13	58.63
3	80	2	100	85.3	85.62	84.82
7	50	1.25	60	63.2	64.38	64.66
7	50	1.25	60	66.9	64.38	64.66
7	50	1.25	60	64.2	64.38	64.66

**Table 5:** ANOVA results and determination coefficients for the BBD-RSM model developed for the treatment process.

Source	Sum of squares	Mean square	F value	*p*-value Prob > F
Model	2225.231	278.1538	94.59664	< 0.0001
A-pH	522.7222	522.7222	177.7713	< 0.0001
B-Time	496.125	496.125	168.7259	< 0.0001
C-Adsorbent dose	840.5	840.5	285.8435	< 0.0001
D-RR198 Con.	108.045	108.045	36.74475	< 0.0001
AB	21.16	21.16	7.196252	0.0147
AD	23.04	23.04	7.835616	0.0114
A^2	40.40358	40.40358	13.74075	0.0015
D^2	26.6438	26.6438	9.061224	0.0072
Residual	55.86798	2.94042		
Lack of fit	48.45798	3.028623	1.226163	0.4961
R^2^ = 0.9755	Adj R^2^ = 0.9652		Pred R^2^ = 9436

**Figure 4:**
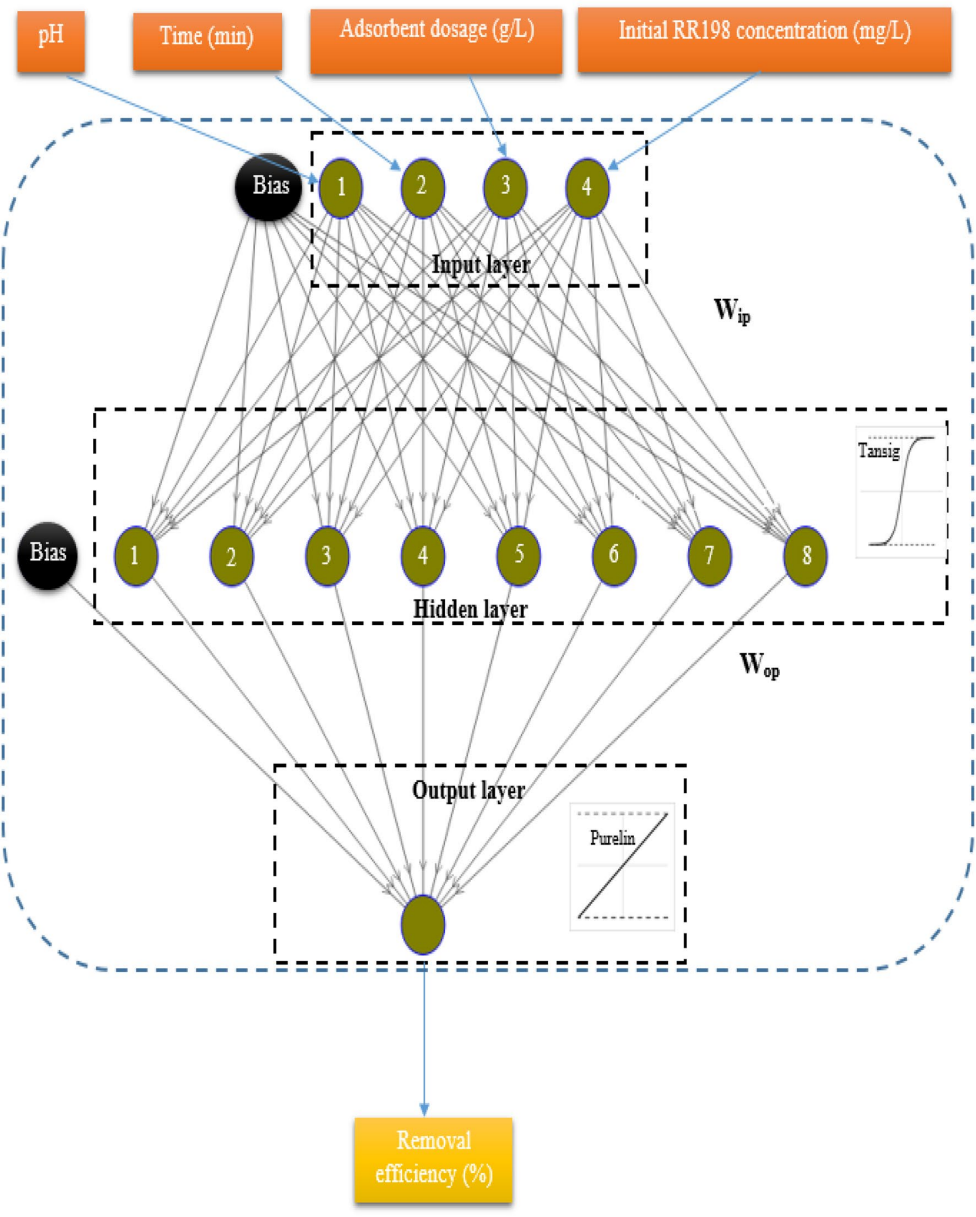
The neural network architecture for a three-layer ANN model with 4-5-1 topology.

**Figure 5:**
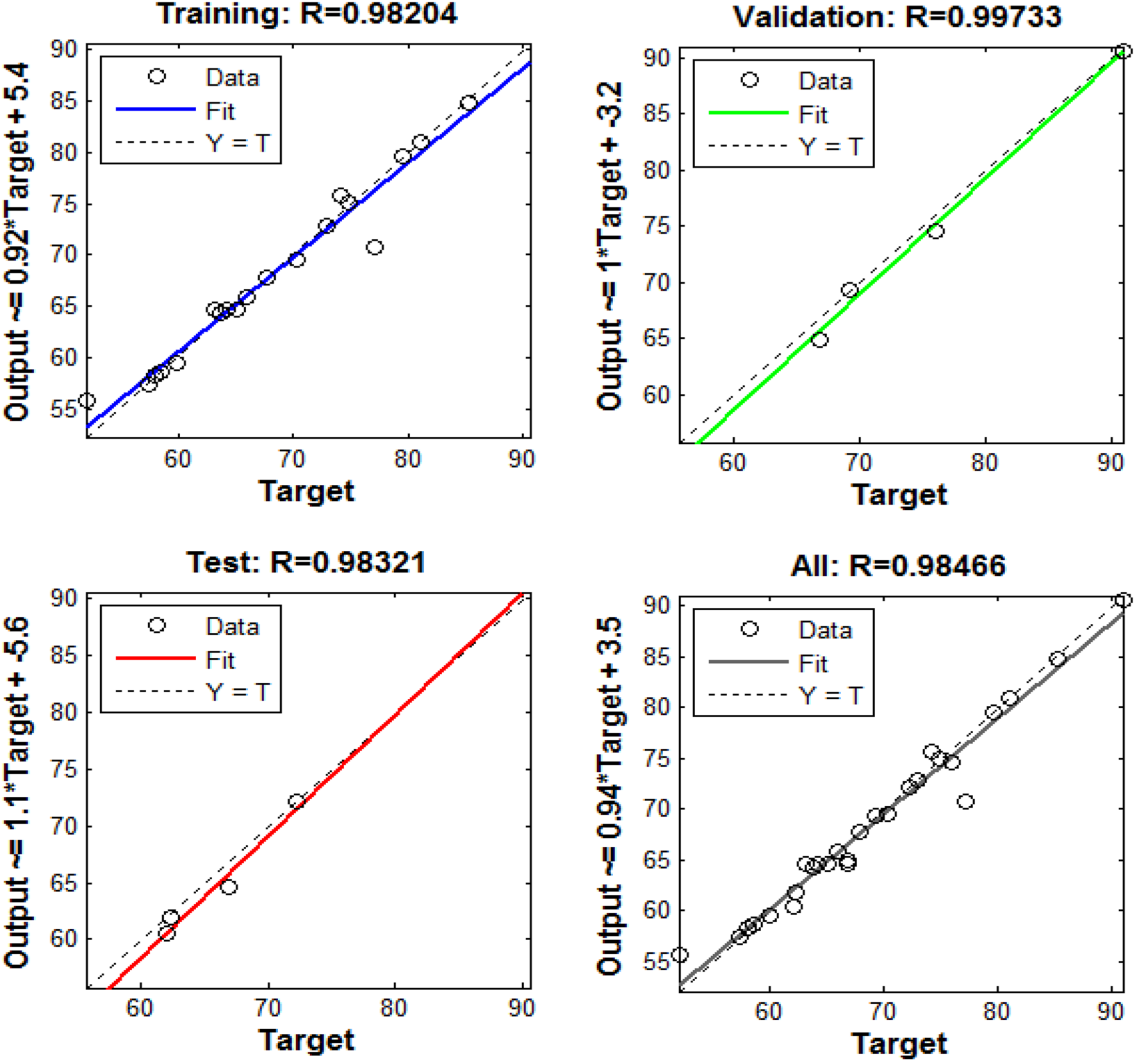
Regression plots for training, testing, and validation, and all data of the three-layer ANN model.

The dataset in Table 4 was also modeled using the ANN method. As before stated, the number of hidden neurons was varied from 1 to 20 to search for the best number of neurons with the lowest MSE value and highest R^2^ value. The results showed that 8 hidden neurons present the smallest MSE and highest R^2^, which give the robust prediction by the ANN model. Accordingly, 4-8-1 topology (Fig. 4) was chosen as the best architecture of the ANN model. Figure 5 shows the R^2^ values of 0.9820, 0.9973, 0.9832, and 0.9846 for training, validation, test, and all data, respectively. These results demonstrate a close correlation and a satisfactory agreement between the experimental and predicated removal efficiency so that this model predicts experimental data with very high accuracy^24^. ANN model predictions are presented in Table 4. ANN model can be expressed as Eq. (7) with the weights and biases given in Table 6.

7 $$ {\text{Y}}_{{\text{n}}} = {\text{ f}}_{0} \left\{ {{\text{b}}_{0} + \mathop \sum \limits_{{{\text{k}} = 1}}^{{\text{h}}} \left[ {{\text{w}}_{{\text{k}}} \times {\text{ f}}_{{\text{h}}} \left( {{\text{b}}_{{{\text{hk}}}} + \mathop \sum \limits_{{{\text{i}} = 1}}^{{\text{m}}} \left( {{\text{w}}_{{{\text{ik}}}} {\text{X}}_{{{\text{ni}}}} } \right)} \right.} \right]} \right\} $$

where Y_n_ presents predicated value, f_0_ transfer function (here, tangent sigmoid) associated with output layer, b_0_ bias of output layer, w_k_ the weights of the output layer, f_h_ shows transfer function (here, purelin) in the hidden layer, b_hk_ bias associated with the hidden layer, w_ik_ the weights of the hidden layer, and X_ni_ independent variable.

**Table 6:** Obtained weights and biases of the built ANN model for the RR198 adsorption by MSE compost ash.

Input (4)-hidden layer (8)					Hidden layer (8)-output (1)	
Weights				Bias	Weight	Bias
0.049364	1.8911	1.3882	− 0.85547	0.26853	− 2.7228	0.25515
− 0.83297	1.9481	− 1.2487	1.217	− 0.047469	2.087	
− 1.1795	− 2.4692	0.68868	1.3754	− 0.041984	1.1439	
1.1868	− 1.468	1.2176	0.95268	0.09805	− 1.3409	
− 0.80077	− 2.5144	0.54167	− 0.32138	− 0.1717	0.47525	
1.5611	1.6247	1.2859	− 0.82074	− 0.27311	− 0.84014	
1.1779	1.7681	− 0.84595	− 1.1771	0.052601	− 0.090767	
− 1.9679	1.0206	1.2434	0.86506	0.14268	− 0.53357	

Prediction precision of the BBD-RSM and ANN developed models are assessed by comparing the R^2^ (as criteria for describing the strength and direction of predicated and experimental values) and MSE (revealing error distribution and the magnitude of errors) values of the ANN and BBD-RSM model and model. R^2^ and MSE were calculated as 0.9755 and 0.0791 for the BBD-RSM model and 0.9846 and 0.0259 for the ANN model. Although statistical criteria show relatively better values for the ANN model compared with the BBD-RSM model, both the models manifested that an excellent prediction ability of experimental data, thus, can be used for modeling and recognizing the behavior of the adsorption process.

### Univariate optimization of the adsorption process

**Figure 6:**
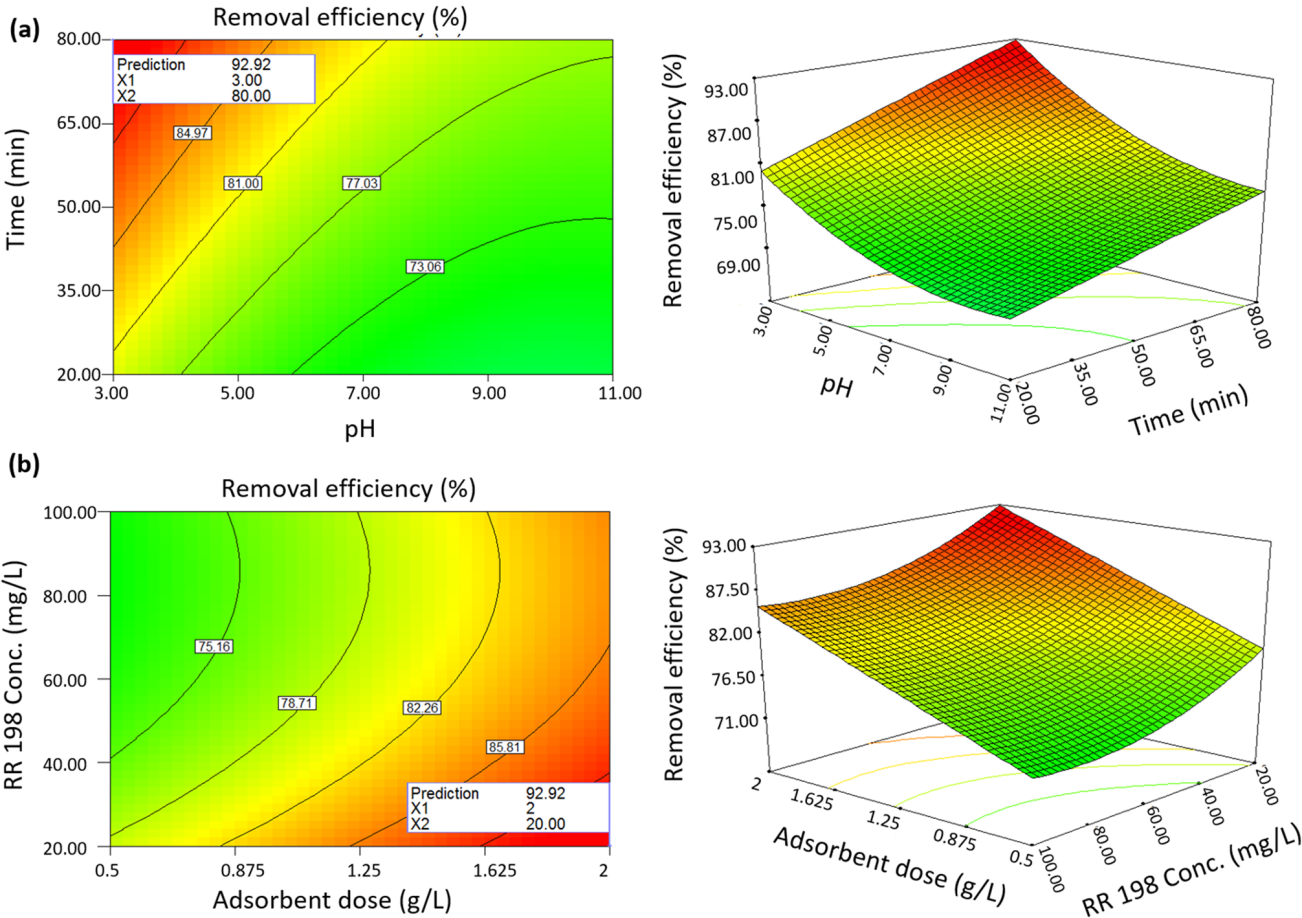
3D response surface plot and contour plot of the absorption of RR198 onto MSW compost ash as a function of pH versus time (**a**) and adsorbent dose versus RR198 Con. (**b**).

**Figure 7:**
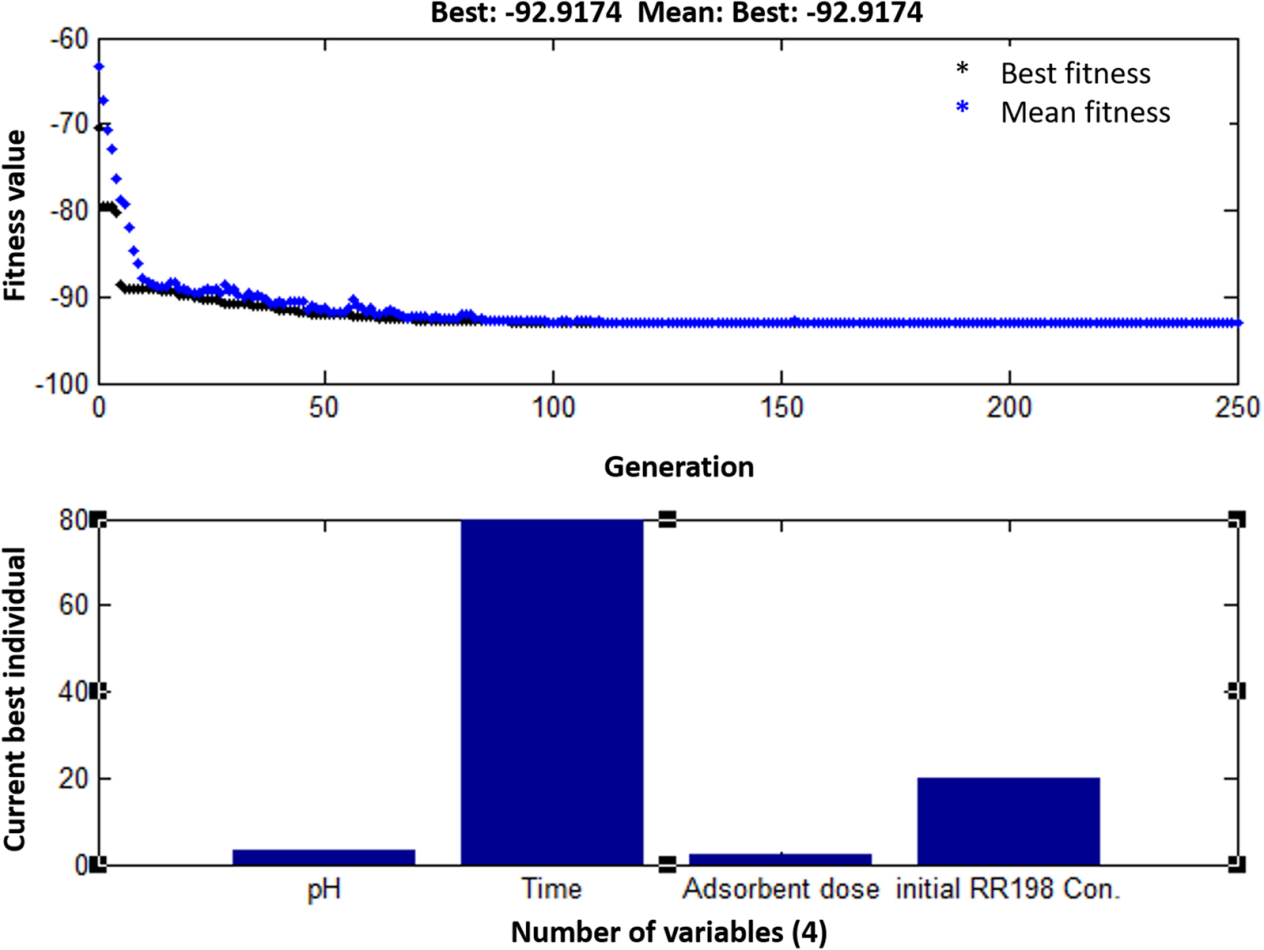
Optimization results of the adsorption process using the GA-ANN approach.

BBD-RSM and GA-ANN methods were used for optimizing the corresponding models in order to explore the levels of the input factors that maximize removal efficiency. The optimization results of the second-order polynomial regression model based on the BBD-RSM method have been presented in Fig. 6. It can be observed that the highest removal efficiency of 92.92% is obtained at solution pH 3, contact time 80 min, adsorbent loading 2 g L^−1^, and initial RR198 concentration 20 mg L^−1^. The developed ANN model was optimized by the GA tool. Figure 7 displays that the generation of solutions at around 100 repetitions showed no improvement in the fitness value and consequently was stopped at 250 repetitions. The current best individual plot demonstrates that the optimal levels of the input variables are consistent with those obtained from the BBD-RSM method, so that that the maximum removal efficiency (92.9%) has been achieved at pH = 3, contact time = 80 min, adsorbent loading = 2 g L^−1^, RR198 concentration = 20 mg L^−1^. The control experiments at the obtained optimum condition were conducted, and the experimental removal efficiency was 93.2% (± 1.2). This confirms a promising capability of both the BBD-RSM and GA-ANN methods for the optimization of the adsorption parameters.

### Influence of parameters

3D and contour plots were used to display variations of the removal efficiency of RR198 by MSW compost ash as a function of the independent variables. Figure 6a shows the effect of the initial pH solution on the adsorption capability of the adsorbent. Results revealed that by increasing the initial solution pH, RR198 uptake by MSW compost ash starts to drop. The highest adsorption efficiency was obtained at initial solution pH of 3. Removal efficiency declined from 92.92 to 77.44% by varying pH from 3 to 11 at the condition of contact time = 80, adsorbent loading = 2 g L^−1^ and initial RR198 concentration = 20 mg L^−1^. This result can be associated with producing repulsive electrostatic force of the adsorbent surface with dye molecules in the basic condition of the solution and attractive electrostatic force in acidic conditions^29^.

The effectiveness of contact time on adsorbent performance is visible in Fig. 7a. It is verified that the adsorbent shows higher removal efficiencies at the prolonged contact time. By rising contact time from 20 to 80 min, the adsorption efficiency showed a 13% increase at the condition of pH = 3, adsorbent loading = 2 g L^−1,^ and initial RR198 concentration = 20 mg L^−1^. Increasing removal efficiency at the higher contact time is likely due to the more opportunity and higher chance to collide adsorbent and dye molecules. The rapid RR98 uptake into the adsorbent in the early contact time comes from the fact that more adsorbent surface is available for the solute to be adsorbed at the earlier contact time, while increased saturation of the adsorbent by dye molecules at the longer contact time causes that the adsorption rate is enhanced more slowly. A similar observation was reported in the literature^8^.

Figure 6b exhibits the impact of the adsorbent loading and initial RR198 concentration on adsorption efficiency. As can been seen, removal efficiency is favored by the higher adsorbent dosage. Removal efficiency showed an increasing rate from 79.25 to 92.92% with the increase of the adsorbent dose from 0.5 to 2 g L^−1^ at the condition of pH = 3, contact time = 80 g L^−1^_,_ and initial RR198 concentration = 20 mg L^−1^. It seems that by enhancing adsorbent dosage at the fixed pollutant concentration, a more active surface will be available for adsorption. On the contrary, the adsorption capacity was lessened at the higher adsorbent dosage owing to the improved chance of the collision between the adsorbate and adsorbent^30^.

It is observed in Fig. 6b that at a higher initial RR198 concentration, removal efficiency rises. The results show that under e condition of pH = 3, contact time = 80 g L^−1^, and adsorbent loading = 2 g L^−1^, adsorption efficiency is elevated from 85.62 to 92.92% by reducing initial RR198 concentration from 100 to 20 mg L^−1^. This comes from the fact that by increasing the initial RR198 concentration at the constant adsorbent dosage, the accessible active surface for the adsorption process will be dropped, leading to dropping the removal efficiency^31^.

### Isotherm studies

Adsorption type, adsorbent surface properties, adsorption capacity, and equilibrium relationship between adsorbent and adsorbate can be identified by designing proper adsorption isotherms^32,33^. For this purpose, linear and nonlinear forms of Freundlich, Langmuir, Temkin, and Dubinin-Radushkevich isotherms were taken to evaluate RR198 adsorption onto the MSW compost ash. Langmuir isotherm model figures out a monolayer adsorption phenomenon of sorbate onto the adsorbent surface. Assumptions for the model include (a) no reaction takes place among sorbate molecules during adsorption, (b) adsorption energy is supposed to be constant onto the adsorbent surface, (c) single layer adsorption appears, and (d) maximum adsorbed amount is dependent on the saturability of adsorption layer^34^. The nonlinear Langmuir adsorption model is expressed as follows:

8 $$ q_{e} = \frac{{q_{m} K_{L} C_{e} }}{{1 + K_{L} C_{e} }} $$

The linear form for Eq. (8) is written as Eq. (9)^5^:

9 $$ \frac{1}{{{\text{qe}}}} = \frac{1}{{{\text{q}}_{{\text{m}}} }} + \frac{1}{{{\text{K}}_{{\text{L}}} {\text{q}}_{{\text{m}}} }}\left( {\frac{1}{{{\text{Ce}}}}} \right) $$

**Figure 8:**
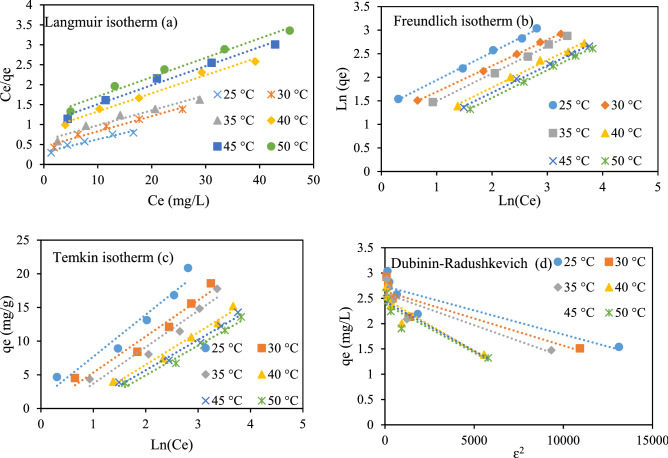
Linear isotherm models for RR 198 adsorption by MSW compost ash.

**Figure 9:**
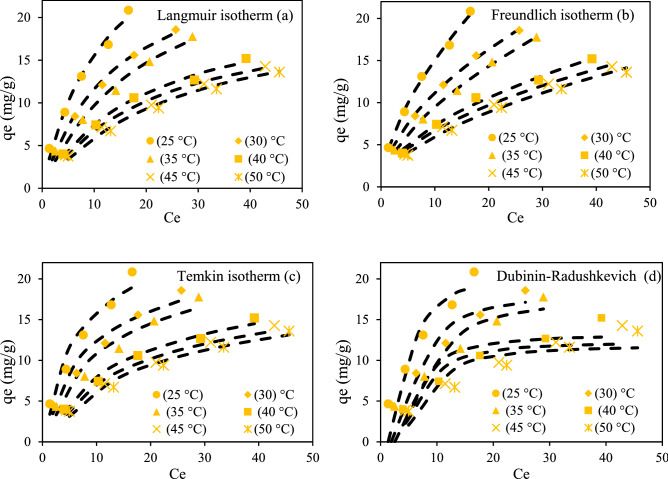
Nonlinear isotherm models for RR 198 uptake by MSW compost ash.

Here, Ce signifies RR198 dye concentration at equilibrium (mg L^−1^), qe is equilibrium adsorption capacity (mg L^−1^), q_m_ is the maximum adsorption capacity (mg g^−1^), K_L_ is the Langmuir adsorption constant (L mg^−1^). By plotting a linear graph between y = 1/q_e_ versus x = 1/C_e,_ the values of q_max_ and k_L_ are obtained from intercept and slope, respectively (Fig. 8a). Also, these parameters were calculated by the nonlinear form of Langmuir isotherm (Fig. 9a). The maximum adsorption capacity was obtained to be within the range of 30.96 mg L^−1^ to 20.619 by changing the solution temperature from 25 to 50 °C, for both nonlinear and linear Langmuir isotherm models. The fitness of data with the isotherm model was checked with statistical metrics of R^2^, Root means square errors (RMSE), and Chi-square (X^2^). The RMSE and X^2^ are calculated according to Eqs. (10) and (11).

10 $$ \sqrt {\frac{1}{n - 1}\mathop \sum \limits_{i = 1}^{n} \left( {\% R_{pred}^{i} - \% R_{exp}^{i} } \right)^{2} } $$

11 $$ X^{2} = \mathop \sum \limits_{i = 1}^{n} \frac{{\left( {\% R_{pred}^{i} - \% R_{exp}^{i} } \right)^{2} }}{{\% R_{exp}^{i} }} $$

As given in Tables 7 and 8, the value of R^2^ at the studied solution temperatures was found higher value, and the values of RMSE and X^2^ were found to lower values in nonlinear Langmuir isotherm when compared to its linear form. The results confirm that nonlinear Langmuir isotherm is superior over linear form to fit equilibrium data of the adsorption process.

**Table 7:** Information of linear isotherm models for RR 198 adsorption onto MSW compost ash.

Tem	Linear Langmuir isotherm					Linear Freundlich isotherm				
	K_L_ (L mg^−1^)	q_m_ (mg g^−1^_)_	R^2^	RMSE	X^2^	n (L g^−1^)	K_F_ (mg g^−1^)	R^2^	RMSE	X^2^
25	0.105	30.960	0.944	0.877	0.399	1.684	3.837	0.9975	0.382	0.050
30	0.086	26.110	0.963	0.706	0.355	1.812	3.122	0.999	0.195	0.015
35	0.063	26.525	0.949	0.699	0.334	1.720	2.499	0.9987	0.173	0.017
40	0.052	21.978	0.987	0.369	0.069	1.730	1.871	0.9911	0.393	0.096
45	0.046	20.921	0.985	0.390	0.088	1.746	1.696	0.9987	0.199	0.021
50	0.040	20.619	0.983	0.394	0.107	1.706	1.479	0.9988	0.173	0.013

Tem.	Linear Temkin isotherm					Linear Dubinin–Radushkevich				
	B_1_	K_T_ (L g^−1^)	R^2^	RMSE	X^2^	q_s_	K_D_	R^2^	RMSE	X^2^
25	6.225	1.267	0.9426	1.366	0.938	15.445	0.0001	0.8112	3.235	5.112
30	5.369	0.993	0.9548	1.062	0.655	13.999	0.0001	0.8046	2.893	4.927
35	5.413	0.727	0.9474	1.091	0.687	13.414	0.0001	0.8041	2.842	4.797
40	4.797	0.524	0.9824	0.522	0.166	12.130	0.0002	0.8595	1.931	2.075
45	4.523	0.475	0.9792	0.529	0.182	11.419	0.0002	0.8524	1.822	1.891
50	4.421	0.413	0.9756	0.546	0.223	10.860	0.0002	0.8393	1.772	2.010

**Table 8:** Information of non-linear isotherm models for RR 198 adsorption onto MSW compost ash.

Tem.	Non-linear Langmuir isotherm					Non-linear Freundlich isotherm				
	K_L_ (L mg^−1^)	q_m__(_mg g^−1^_)_	R^2^	RMSE	X^2^	n (L g^−1^)	K_F_ (mg g^−1^)	R^2^	RMSE	X^2^
25	0.105	30.960	0.9854	0.772	0.351	1.652	3.743	0.9955	0.393	0.050
30	0.086	26.110	0.9893	0.616	0.310	1.809	3.118	0.9985	0.196	0.015
35	0.063	26.525	0.9884	0.603	0.289	1.679	2.406	0.9987	0.198	0.017
40	0.052	21.978	0.992	0.361	0.064	1.850	2.099	0.9902	0.459	0.099
45	0.046	20.921	0.9913	0.344	0.072	1.881	1.971	0.9976	0.256	0.065
50	0.040	20.619	0.994	0.307	0.072	1.770	1.632	0.9983	0.319	0.061

Tem.	Non-linear Temkin isotherm					Non-linear Dubinin–Radushkevich				
	B_1_	K_T_ (L g^−1^)	R^2^	RMSE	X^2^	q_s_	K_D_	R^2^	RMSE	X^2^
25	398.001	1.267	0.9426	1.366	0.938	20.415	0.009	0.9243	2.425	3.634
30	469.242	0.993	0.9548	1.062	0.655	18.170	0.016	0.9242	2.284	3.290
35	473.111	0.727	0.9474	1.091	0.687	17.495	0.024	0.9164	2.229	3.497
40	542.540	0.524	0.9824	0.522	0.166	13.164	0.014	0.8176	1.755	1.725
45	584.562	0.475	0.9792	0.529	0.182	12.273	0.015	0.7961	1.706	1.585
50	594.331	0.401	0.9756	0.555	0.247	11.830	0.019	0.7896	1.666	1.615

Freundlich isotherm was originally developed to explain heterogeneous multilayer adsorption. Also, an energy reduction is assumed to occur in adsorbing sites and intermolecular reaction among adsorbed molecules for the adsorption process, which follows Freundlich isotherm^5^. For the Freundlich adsorption model, nonlinear and linear forms were expressed as follows:

12 $$ {\text{q}}_{{\text{e}}} = {\text{K}}_{{\text{f}}} C_{e}^{1/n} $$

13 $$ {\text{Ln q}}_{{\text{e}}} = {\text{Ln K}}_{{\text{f}}} + \frac{1}{{\text{n}}}{\text{LnC}}_{{\text{e}}} { } $$

K_f_ stands for the Freundlich constant (mg g^−1^), presenting adsorption capacity, 1/n describes the exponent of non-linearity. n is the Freundlich constants. Overall, n < 1 signifies poor adsorption, n = 1–2 indicates average adsorptions and n = 2–10 is obtained for good adsorptions. Thealues of n and k_f_ are obtained by the linear graph between log (C_e_) versus log (q_e_) or from drawing a nonlinear form of Eq. (13) (Figs. 8b, 9b). The fitness of the isotherm model to equilibrium data was evaluated with statistical metrics of R^2^, RMSE and X^2^. As given in Tables 7 and 8, nonlinear Langmuir isotherm had larger R^2^ values and lowered RMSE and X^2^ values at the studied solution temperatures than its linear form. As a result, the linear Freundlich isotherm was found superior over the nonlinear form in terms of fitness to equilibrium data.

Temkin adsorption isotherm assumes that the heat of adsorption of all the molecules drops linearly with coverage, due to due to adsorbent–adsorbate interactions. The derivation of the Temkin isotherm reveals a linear heat loss of sorption rather than logarithmic, as implied in the Freundlich model^34^. The nonlinear form of the Temkin isotherm model is given as^35^:

14 $$ {\text{q}}_{{\text{e}}} = \frac{RT}{{b_{T} }}{\text{lnK}}_{T} {\text{C}}_{{\text{e}}} { }{\text{.}} $$

Equation (15) shows the linearized form of Eq. (14).

15 $$ {\text{q}}_{{\text{e}}} = \frac{RT}{{b_{T} }}{\text{ lnK}}_{{\text{T}}} + \frac{RT}{{b_{T} }}{\text{lnC}}_{{\text{e}}} $$

R is the universal gas constant (8.314 J mol^−1^ K^−1^), K_T_ shows Temkin itherm constant (L g^−1^), B_T_ is the Temkin constant corresponding to the heat of the adsorption (J mol^−1^), and T is the temperature in Kelvin. b and K_T_ values are obtained from the slope and intercept of the linear graph of x = ln (C_e_) versus y = q_e_ (Fig. 8c). These parameters were also calculated by a nonlinear form of Temkin isotherm (Fig. 9c). The information related to calculated coefficients has been given in Tables 7 and 8. Also, the values of R^2^, RMSE, and X^2^ to evaluate the finesse of linear and nonlinear Temkin isotherm are presented in Tables 7 and 8, respectively. These parameters proved that both linear and nonlinear forms of Temkin isotherm appear almost similar fitness values.

In general, Dubinin–Radushkevich isotherm model was developed as an empirical model to predict the physical or chemical nature of the adsorption process. It shows successful adsorption in liquid-solid heterogeneous systems. Dubinin–Radushkevich model than Langmuir is considered more general as constant sorption potential, and derivation homogenous surface in the isotherm is not assumed. The nonlinear Dubinin–Radushkevich isotherm model is written as^36,37^.

16 $$ {\text{q}}_{{\text{e}}} = q_{m} exp^{{\beta \varepsilon^{2} }} $$

Equation (17) shows the linearized form of Eq. (16).

17 $$ {\text{lnq}}_{{\text{e}}} = {\text{ lnq}}_{{\text{s}}} - K_{D} \varepsilon^{2} $$

where q_m_ stands for the adsorption capacity (mg g^−1^) based on the Dubinin-R monolayer adsorption approach, β shows sorption energy, and ε is the Polanyi potential representing the equilibrium concentration. It is calculated as:

18 $$ \varepsilon = RTln\left( {1 + \left( {\frac{1}{{C_{e} }}} \right)} \right) $$

By drawing ln q_e_ via $\varepsilon^{2}$, the slope and intercept of the plot give K_D_ and qs values (Fig. 8d). These parameters were also calculated from the nonlinear isotherm form (Fig. 9d). The nonlinear form of the model demonstrates a more accurate fitness value than the linear form, according to R^2^, RMSE, and X^2^ parameters (Tables 7, 8). Dubinin–Radushkevich isotherm shows a successful application for the determination of the physical or chemical nature of sorption. For this purpose, Equation (19) is used to calculate the parameter of E.

19 $$ E = \frac{1}{{\sqrt {2K_{D} } }} $$

E < 8 kJ mol^−1^ indicates physical adsorption, and the values ranging from 8 to 16 kJ mol^−1^ describe chemical adsorption. The value of E calculated for the adsorption process based on nonlinear Dubinin–Radushkevich isotherm was below 8 kJ mol^−1^, suggesting the physical nature of RR198 adsorption onto the MSW compost ash.

As initially stated, the findings related to the different isotherm models fitted to experimental data are given in Tables 7 and 8. According to the calculated values of statistical metrics of R^2^, RMSE and X^2^, the equilibrium data are better described by both linear and nonlinear Freundlich isotherm, as compared with Langmuir, Temkin, and Dubinin–Radushkevich isotherm models.

### Kinetic studies

**Figure 10:**
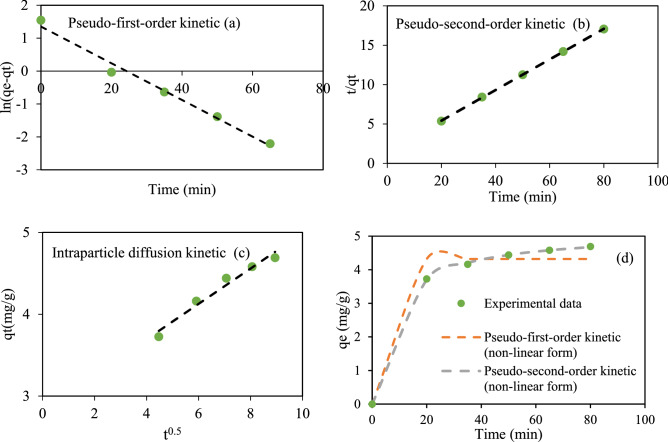
Linear and nonlinear kinetic models for RR 198 adsorption by MSW compost ash.

Kinetic models including pseudo-first-order, pseudo-second-order, and intraparticle diffusion models were applied to explore the best-fitted model to the experimental data. The kinetic study helps to describe solute uptake rate, contact time needed for the adsorption process, mechanism of sorption and adsorption constant rate, as well as chemical reactions^7^. The pseudo-first-order kinetic model was tested to fit experimental data^38^. The model is typically developed to study liquid/solid adsorption system, indicating that penetration depends on adsorption capacity, and variation of adsorption amount as a function of time is proportional to unoccupied sites on the adsorbent surface. The nonlinear and linear forms of pseudo-first-order kinetic model are given as^39,40^:

20 $$ Q_{t} = Q_{e } \left( {1 - e^{{ - k_{1} t}} } \right) $$

21 $$ \ln \left( {{\text{q}}_{{\text{e}}} - {\text{q}}_{{\text{t}}} } \right) = {\text{lnq}}_{{\text{e}}} - {\text{K}}_{1} {\text{t }} $$

q_e_ and q_t_ show the adsorption capacities at equilibrium and time t (mg g^−1^), and k_1_ stands for the rate constant (min^−1^), respectively. ln(q_e_) and k_1_ are the intercept and slope of the linear graph of x = t versus y = ln(q_e_−q_t_), respectively. q_e_ and k_1_ values are obtained from a linear graph between y = $\mathrm{ln}\left({\mathrm{q}}_{\mathrm{e}}-{\mathrm{q}}_{\mathrm{t}}\right)$ and x = t (Fig. 10a). Those were also determined from the nonlinear pseudo-first-order kinetic model (Fig. 10d). The information related to parameters of linear and nonlinear forms of the pseudo-first-order kinetic model has been represented in Table 9. A comparative study between linear and nonlinear forms by statistical metrics of R^2^, RMSE, and X^2^ indicated that the linear form is better suited to explain the experimental data compared to the nonlinear form. Table 9 shows R^2^ = 0.9853, RMSE = 0.193, and X^2^ = 0.0245 for linear pseudo-first-order kinetic model and R^2^ = 0.9629, RMSE = 0.346, and X^2^ = 0.148 for its nonlinear form.

**Table 9:** Information of linear and non-linear kinetic models for RR 198 adsorption onto MSW compost ash.

	Pseudo-first-order kinetic					Pseudo-second-order kinetic					Intraparticle diffusion kinetic		q_exp_ (mg g^−1^)
	K_2_ )g mg^−1^ min^−1^)	q_e_ (mg g^−1^)	R_2_	RMSE	X^2^	K_1_ (min^−1^)	q_e_ (mg g^−1^)	R_2_	RMSE	X^2^	K_p_ (mg g^−1^ min^−1/2^)	K_p_ (mg g^−1^ min^−1/2^)	
Linear parameters	0.0558	3.864	0.9853	0.193	0.0245	0.1262	5.144	0.9999	0.0223	0.0006	2.831	0.9669	4.96
Non-linear parameters	1.000	4.319	0.9629	0.346	0.148	0.026	5.119	0.9998	0.0243	0.000753			

Pseudo-second-order kinetic model suggests that adsorption behavior obeys pseudo-second-order reaction with the rate-limiting step. In chemical adsorption, the occupancy rate of adsorption sites has a direct relationship with the square number of unoccupied sites^38^. The nonlinear and linear forms of the kinetic model are written as Eqs. (22) and (23), respectively^39,41^.

22 $$ Q_{t} = \frac{{k_{2} Q_{e}^{2} t}}{{1 + k_{2} Q_{e} t}} $$

23 $$ \frac{{\text{t}}}{{{\text{qt}}}} = \frac{1}{{{\text{k}}_{2} {\text{qe}}^{2} }} + \frac{1}{{{\text{qe}}}}{\text{t }} $$

q_e_ and q_t_ denote the amounts of RR198 adsorbed at equilibrium and time t (mg g^−1^), and k_2_ is devoted to the rate constant (g mg^−1^ min^−1^), respectively. Q_e_ and k_2_ values are determined by a linear graph between y = $\frac{{\text{t}}}{{{\text{qt}}}}$ and x = t (Fig. 10b), and also nonlinear pseudo-second-order kinetic model (Fig. 10d). The linear and nonlinear parameters of the kinetic model are shown in Table 9. Error functions of R^2^, RMSE, and X^2^ for the nonlinear pseudo-second-order kinetic model were calculated as 0.9998, 0.0243, and 0.00075, respectively. Those for linear form were found to be 0.9999, 0.0223, and 0.0006. The results indicate that both linear and nonlinear forms of the kinetic model have similar fitness values to experimental data.

In particle diffusion kinetic model as a commonly used kinetic model was also used in the present study. Four consecutive stages were involved in the kinetic model as follows: (a) transport of sorbate in the bulk solution; (b) film diffusion; (c) mass diffusion migration of sorbate molecules within the pores of sorbent; (d) Adsorption of solute molecules onto adsorbent via chemical reactions like complexation, ion exchange, and chelating reactions^42^. The intraparticle diffusion kinetic model is given as^35^.

24 $$ {\text{q}}_{{\text{t}}} = {\text{K}}_{{\text{p}}} {\text{t}}^{0.5} + {\text{C}} $$

In Eq., q_t_ represents the adsorption capacity at time t in (mg g^−1^), k_pt_ describes the intraparticle diffusion rate constant (mg g^−1^ min^−1/2^). k_p_ value is calculated from the linear graph of x = t^1/2^ versus y = q_t_ (Fig. 10c) and shown in Table 9.

To made a comparison among the different kinetic models in terms of fitness value, the error functions of R^2^, RMSE and X^2^ were applied. As shown in Table 9, the calculated values of R^2^, RMSE, and X^2^ revealed that both the linear and nonlinear form of the pseudo-second-order kinetic model offers the best fit to the experimental equilibrium data when compared with pseudo-first-order and intraparticle diffusion kinetic models.

### Thermodynamic studies

Thermodynamic properties of the RR198 adsorption onto the MSW compost ash were studied under the optimal condition of the process parameters and a temperature of 298, 303, 308, 313, and 318, and 323 °C. Equation (25) was used to determine the $\Delta {\mathrm{G}}^{^\circ }$ thermodynamic parameter and $\Delta {\mathrm{S}}^{^\circ }$ and ${\Delta \mathrm{H}}^{^\circ }$ are obtained from the intercept and slope factor of Eq. (26)^43^. Ke^o^ represents the thermodynamic equilibrium constant (dimensionless).

25 $$ \Delta {\text{G}}^{^\circ } = - {\text{RT ln }}Ke^{^\circ } $$

26 $$ \ln Ke^{^\circ } = \frac{{\Delta {\text{S}}^{^\circ } }}{{\text{R}}} - \frac{{\Delta {\text{H}}^{^\circ } }}{{{\text{RT}}}} $$

27 $$K{e}^{^\circ }= \frac{{K}_{L}^{m}{\left[adsorbate\right]}^{^\circ }}{\gamma }$$

28 $${K}_{L}^{m}= 1000 \times {\mathrm{K}}_{\mathrm{L}} \times \mathrm{ MW}$$

**Figure 11:**
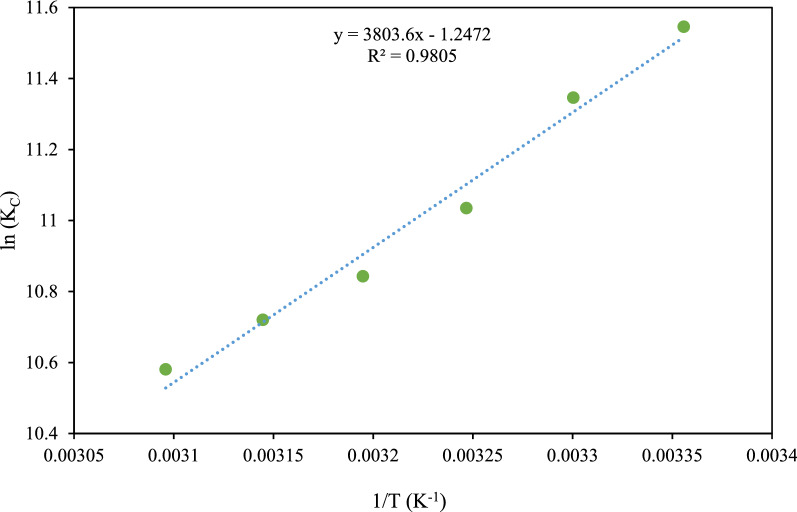
A thermodynamic model for RR 198 adsorption by MSW compost ash.

Here, $\Delta {\text{G}}^{^\circ }$ stands for the change of free Gibbs energy (kJ mol^−1^), R represents the gas constant (8.314 J mol^−1^ K^−1^), T describes the temperature in Kelvin, $\Delta {\text{H}}^{^\circ } { }$ shows enthalpy change (kJ mol^−1^), $\Delta {\text{S}}^{^\circ }$ is entropy change (J mol^−1^ K^−1^), and K_d_ is distribution coefficient, MW is the molecular weight of adsorbate (Reactive red 198; 967.5 g mol^−1^), [adsorbate]^o^ describes the standard adsorbate concentration (1 mol L^−1^), K_L_ is the best-fitted isotherm constant (L mg^−1^), and γ is the activity coefficient (dimensionless). We considered the adsorbate solution very diluted to assume the activity coefficient of unitary (equal to 1). By plotting ${\mathrm{lnK}}_{\mathrm{d}}$ via 1/T, the slope and intercept were used to calculate the values of $\Delta {\mathrm{H}}^{^\circ }$ and $\Delta {\mathrm{S}}^{^\circ }$ respectively (Fig. 11). The obtained thermodynamic information for the adsorption process model is summarized in Table 10. Negative $\Delta {\mathrm{G}}^{^\circ }$ value is an indication of spontaneous adsorption. Further, the less value of $\Delta {\mathrm{G}}^{^\circ }$ at the higher temperatures indicates undesirability of the adsorption process at the elevated temperature. The negative value of ${\Delta \mathrm{H}}^{^\circ }$ suggests the exothermic sorption so that adsorption efficiency is increased at the lower temperatures. The negative $\Delta {\mathrm{S}}^{^\circ }$ value reflected the randomness at the solid/liquid interface^44^.

**Table 10:** Thermodynamic information of RR198 adsorption on MSW compost ash.

Temperature (°K)	K_L_ (L mg^−1^)	K^o^	$\Delta \mathrm{G}(\mathrm{kJ }{\mathrm{mol}}^{-1})$	$\Delta {\mathrm{S}}^{^\circ }(\mathrm{J }{\mathrm{mol}}^{-1} {\mathrm{K}}^{-1})$	${\Delta \mathrm{H}}^{^\circ } (\mathrm{kJ mo}{\mathrm{l}}^{-1})$	R^2^
298	0.0017	1598.3	− 28.414	− 10.369	− 31.623	0.9805
303	0.0018	1750.2	− 28.343			
308	0.0017	1624.4	− 28.217			
313	0.0019	1789.9	− 28.207			
318	0.0019	1819.9	− 28.183			
323	0.0018	1712.5	− 28.106			

### Reusability test of MSW compost ash

**Figure 12:**
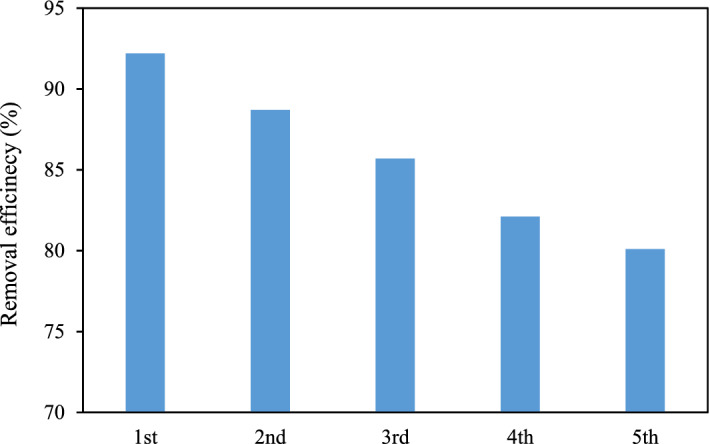
Reusability test for the MSW compost ash.

The recovery of adsorbent was considered a very important feature in the present study because of its practical application and economic feasibility. In this context, reusability study of the adsorbent was carried out for successive five cycles, where after each run, the adsorbent was separated from the medium, dissolved in 0.1 mol L^−1^ NaOH solution for 3 h, and finally rinsed by deionized water to isolate form the adsorbate^7^. The relevant results, as given in Fig. 12, show that the adsorption efficiency of MSW compost ash has maintained over 80%. So, it confirms the promising reusability of MSW compost ash. The slight decline of removal efficiency can be assigned to the decrease in the available active surface area of the adsorbent due to the trapped dye molecules in the adsorbent pores even after frequent washing of the adsorbent and the mass loss of the adsorbent after washing operation.

### Comparison with other adsorbents

In the present study, MSW compost ash as a low-cost and efficient adsorbent was used for RR 198 adsorption from an aquatic matrix, showing a remarkably good performance in the adsorption process. To further signify its efficiency, the prepared adsorbent was compared with some other reported natural adsorbents with respect to their adsorption capacity for dye pollutants in an aqueous solution. Table represents the maximum adsorption capacity for different natural adsorbents. As seen, MSW compost ash has superiority in dye adsorption when compared with some given adsorbents in Table 11. As a result of reasonable adsorption capacity, low cost and abundance, availability, it can be proposed as an attractive alternative for dye’s removal.

**Table 11:** Comparison of dye adsorption efficiency of different adsorbents.

Adsorbents	Dyes	Maximum adsorption capacity (mg g^−1^)	References
Fly ash	Crystal violet	4.73	^45^
	Rosaniline hydrochloride	4.81	
Carbon slurry	Ethyl orange	198.4	^46^
	Metanil yellow	211.9	
	Acid blue 113	221.2	
Carbon slurry	Chrysoidine G	80.6	^47^
	Crystal violet	163.0	
	Meldola blue	171.2	
Red Mud	Rhodamine B	5.5	^48^
	fast green	7.5	
	methylene blue	16.7	
Boron Industry Waste	Acid Red 183	48.5	^49^
	Reactive Blue 4	46.7	
Sugar cane dust	Malachite Green	4.879	^50^
	Rhodamine B	4.261	
Neem sawdust	malachite green	4.354	^51^
Orange peel	Acid violet 17	19.88	^52^
Flyash	Methylene blue dye	3.074	^53^
Activated carbon		9.813	
Flyash	Congo red dye	4.125	
Activated carbon		15.80	
Clinoptilolite	Basic Yellow 28	59.6	^54^
Amberlite XAD-4		8.7	
MSW compost ash	Reactive red 198	30.9	This study

## Conclusion

In the present study, MSW compost ash was characterized by using several methods, and its capacity in the adsorptive removal of RR198 from synthetic wastewater was studied and optimized. BBD-RSM approach developed a quadratic polynomial regression model with F-value = 94.596 and R^2^ = 0.9436, and ANN suggested a three-layer model with test-R^2^ = 0.9832, the structure of 4-8-1 and learning algorithm type of Levenberg–Marquardt backpropagation. By comparing the two modeling approaches, the ANN model can be introduced as a more reliable model to predict the responses closer to the experimental results. The same optimization results were achieved by BBD-RSM and GA-ANN techniques. The maximum removal efficiency of RR198 (92.8%) was observed under the condition of pH = 3, contact time = 80 min, RR198 = 20 mg L^−1^ and MSW compost ash loading = 2 g L^−1^. The adsorption behavior was more in line with Freundlich isotherm, pseudo-second-order kinetic models, which demonstrate multilayer adsorption with a heterogeneous system and heterogeneous chemical adsorption on the adsorbent surface, respectively. Also, the thermodynamic study indicated the exothermic nature of the RR198 adsorption onto MSW compost ash. The reusability test of the adsorbent shows no obvious decline of removal efficiency after five successive cycles of reuse. In conclusion, MSW compost ash as an economical, reusable and efficient adsorbent can be proposed to be applied in the adsorption process for dye pollutants removal from aquatic environments, and both BBD-RSM and ANN approaches are highly potential methods for modeling the adsorption process. This study also provides preliminary information, which is helpful for developing the adsorption process on an industrial scale.
